# Preliminary feasibility and hemodynamic performance of a newly-developed self-expanding bioprosthesis and 16-F delivery system in transcatheter aortic valve implantation in sheep

**DOI:** 10.7555/JBR.26.20120011

**Published:** 2012-05-17

**Authors:** Jing Cai, Yanhui Sheng, Shijiang Zhang, Wei Sun, Rong Yang, Liping Miao, Xiangqing Kong

**Affiliations:** a Department of Cardiology,; b Department of Cardiac Surgery, the First Affiliated Hospital, Nanjing Medical University, Nanjing, Jiangsu 210029, China.

**Keywords:** percutaneous, self-expanding bioprothesis, aortic valve, sheep

## Abstract

We sought to evaluate the feasibility and hemodynamic performance of a new self-expanding bioprosthesis and 16-F delivery system in sheep. A 23-mm new self-expanding aortic bioprosthesis was implanted in sheep (*n* = 10) with a 16-F catheter *via* the right common carotid artery. Each sheep underwent angiography and coronary angiography before intervention, immediately and 1 h after stent implantation. Electrocardiographic monitoring was carried out during and 2 h after the procedure. Transthoracic echocardiography was employed to detect hemodynamic performance before intervention, immediately and 1 and 2 h after stent implantation. All sheep were euthanized 2 h after successful implantation for macroscopic inspection. In all cases, the new self-expanding aortic bioprosthesis was successfully delivered to the aortic root and released with a 16-F catheter. Successful implantation was achieved in 8 of 10 sheep. Hemodynamic performance and device position of successful implantation were stable 2 h after device deployment. Atrioventricular block was not observed. We conclude that it is feasible to implant the new self-expanding aortic valve with a 16-F delivery system into sheep hearts via the retrograde route.

## INTRODUCTION

With increasing life expectancy and an aging population, the number of patients with aortic valve stenosis (AS) will increase. The prevalence of AS is 4.6% in adults aged ≥75 years[1]. However, a significant proportion of patients with symptomatic severe AS are denied or not offered surgery due to high surgical risk or non-operability for chest-opening replacement of the aortic valve[2]-[5]. After emerging as an alternative to the treatment of severe aortic stenosis, transcatheter aortic valve implantation (TAVI) has become a possibility to such selected patients. There are two major types of aortic valve stents: the balloon-expandable bioprosthesis and the self-expanding bioprosthesis. In the latter, the self-expanding revalving system has converted the procedure to a completely percutaneous one with the creation of a 23-mm prosthesis with a 18-F delivery system. However, many patients do not have the opportunity to have interventional valve implantation because of certain arterial conditions (e.g., stenosis, tortuosity, aneurysm, and narrowing >70%). Furthermore, many complications associated with the procedure [e.g., obstruction of the coronary artery, paravalvular leaks, and atrioventricular (AV) block] remain for patients undergoing TAVI. Among these complications, AV block caused by bundle injury and subsequent pacemaker implantation deserve attention. Possible causes of this complication include mechanical pressure and tissue injury derived from the radial force of the lower-third portion of the device on conductive bundles[6]. To improve the procedure and to reduce the prevalence of complications, we developed a new self-expanding revalving system (Venus A-valve, Hangzhou, China) with a relatively smaller lower one-third portion and a 16-F delivery system, which may cause less injury to the arteries and conductive bundles after implantation. The feasibility and hemodynamic performance of this device in animal models has not been evaluated. There is no representative chronic model of aortic stenosis using animals, so we undertook TAVI with this 16-F self-expanding bioprosthesis in healthy sheep to preliminarily validate its feasibility and hemodynamic safety.

## MATERIALS AND METHODS

### Preparation of animals

The study protocol was approved by the Ethics Committee of the First Affiliated Hospital of Nanjing Medical University, Nanjing, Jiangsu, China. All animals received humane care in compliance with the “Guide for the Care and Use of Laboratory Animals” issued by Ministry of Science and Technology of the People's Republic of China. Ten healthy sheep (8 males and 2 females) weighing 45.7±2.18 kg (range, 42.3-48.4 kg) were used. General anesthesia was induced with ketamine (15 mg/kg, i.m.) followed by 10 mL of 3% pentobarbital sodium (i.v.). All procedures were undertaken in the supine position using a V-shaped board to support the sheep's back. Tracheal intubation and mechanical ventilation were employed to ensure the stability of vital signs, which were monitored during the entire procedure, together with electrocardiography (ECG). Transthoracic echocardiography (TTE) was done with an Hp CX50 system equipped with a X5-1 pure-wave transducer (1-5 MHz) before intervention, throughout the whole procedure, and at follow-up.

### Implantation of valved stents

**Fig. 1:**
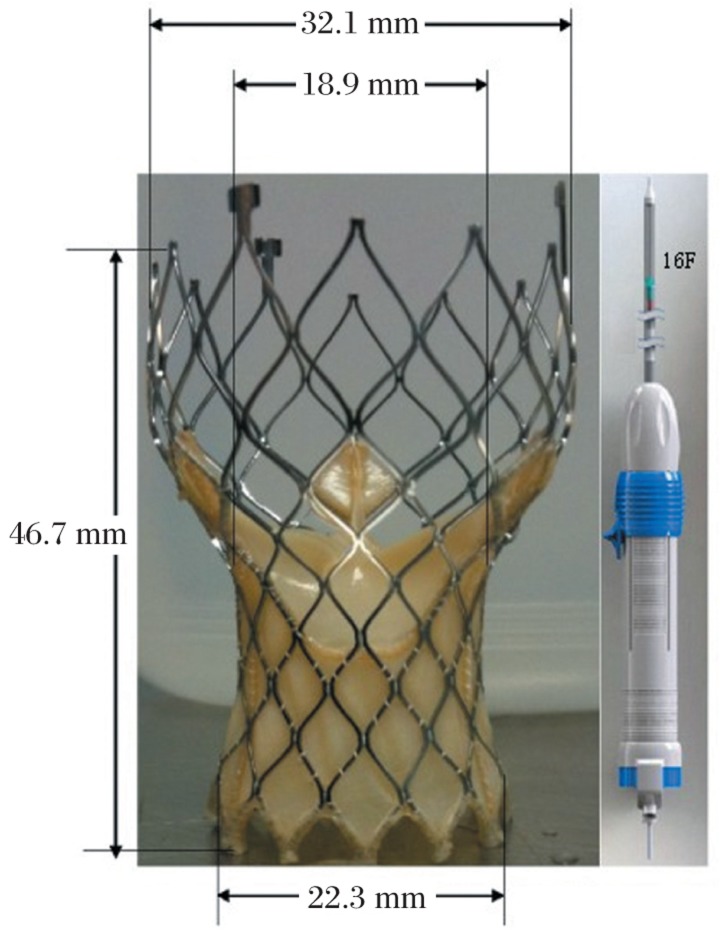
The design of improved self-expanding bioprosthesis and its 16-F delivery system.

The new self-expanding revalving system (Venus A-valve, Hangzhou, China) is shown in ***Fig. 1***. The bioprosthesis was manufactured by suturing valve leaflets and a skirt, made from a single layer of porcine pericardium, into a tri-leaflet configuration. The self-expanding, multi-level frame was made of Nitinol and was radiopaque. Arterial access was obtained with a standard surgical cutdown of the right common carotid artery. A 6-F pigtail catheter was introduced into the ascending aorta via the right femoral artery for aortography to detect the exact position of the native aortic valve as well as the anatomic parameters of its aortic valvular complex. The delivery route was established by introducing a 0.035-inch superstiff guidewire into the left ventricle via another pigtail catheter from the right carotid artery. After the pigtail catheter was removed, a 16-F delivery sheath equipped with the 23-mm aortic self-expanding bioprosthesis was advanced into the left ventricle. When confirmed by fluoroscopy and TTE that the stent had been delivered to the optimal position, the device was released and the delivery sheath retreated. Angiography of the aortic root was carried out again to detect the position of the implanted device, blood flow in the coronary artery, as well as aortic regurgitation. The carotid artery was sutured after the delivery sheath was removed. The pigtail catheter was retained for later angiography.

### Evaluation

All sheep underwent angiography of the aortic root and coronary angiography immediately after stent implantation to assess the position of the device, its function, and its impact on coronary blood flow. ECG monitoring was done to each sheep during and 2 h after the procedure to observe arrhythmias. TTE was employed to detect hemodynamic performance and post-procedural regurgitation immediately and 1 and 2 h after the procedure. The following parameters were evaluated in all experimental animals: the diameter of the left atrium (LA diameter); diameter of the left ventricle in the end-diastolic period (LVDd); diameter of the left ventricle in the end-systolic period (LVDs); ejection fraction of the left ventricle (LVEF) calculated by the Simpson method; mean transvalvular gradient calculated using the Bernoulli formula; the valve effective orifice area (EOA) measured using the continuity equation. The presence, degree, and type (paravalvular versus transvalvular) of aortic regurgitation was recorded in all experimental animals. The degree of AR and mitral regurgitation was classified as: none/trivial, mild, moderate, and severe[7]. All surviving sheep were euthanized 2 h after successful implantation for macroscopic inspection.

### Statistical analysis

Categorical variables are expressed as percentages and continuous variables are summarized by mean±standard deviation (SD). Repeated measure ANOVA was conducted to compare the difference among time-points. Aortic regurgitation level was compared by repeated ANOVA based on rank transformed scale. Multiple comparisons between time-points are conducted by anova postestimation method (Wald test). All statistical analyses were done using STATA 12 (StataCorp, College Station, TX, USA). *P*≤0.05 was considered statistically significant.

## RESULTS

### Characteristics of the sheep

Angiography revealed the mean diameter of the aortic annulus to be 19.56±1.61 mm (range, 16.9-22.6 mm). The mean diameter of the aortic root was 22.10±1.37 mm (19.3-24.0 mm). The diameter of the sinotubular junction was 20.79±1.65 mm (17.2-22.8 mm), and the diameter of the left ventricular outflow tract was 19.94±1.67 mm (17.0-22.8 mm). Animal characteristics and the anatomic data of the aorta are shown in ***Table 1***.

**Table 1:** Animal characteristics and anatomic parameters of the aortic valvar complex

Number	Sex	Weight (kg)	Diameter of the aortic annulus (mm)	Diameter of the aortic root (mm)	Diameter of the sinotubular junction (mm)	Diameter of the left ventricular outflow tract (mm)
1	M	46.9	20.4	22.1	21.2	20.4
2	M	47.2	16.9	19.3	17.2	17.0
3	M	45.5	20.2	21.0	20.8	20.5
4	M	48.4	20.2	23.5	22.8	21.6
5	F	42.3	20.6	21.7	21.4	20.7
6	M	43.1	19.1	23.2	21.5	19.8
7	M	46.9	18.4	21.3	19.3	19.0
8	F	47.8	17.9	22.5	19.9	18.1
9	M	44.3	22.6	24.0	22.6	22.8
10	M	42.8	19.3	22.4	21.2	19.5

### Device implantation

In all cases, the 23-mm self-expanding biopros thesis was successfully delivered to the aortic root and released *via* a 16-F delivery system. Successful implantation was achieved in 8 of 10 sheep. One sheep died within 2 h after the procedure due to partial obstruction of the orifice of the left coronary artery. This was caused by the position of the device being too high. Another sheep showed acute rupture of the mitral chorda tendineae and severe regurgitation caused by the position of the device being too low, leading to cardiac failure. Each case was confirmed by necropsy. During the procedure, all sheep had transient rhythm disturbances, most of which was ventricular premature.

### Follow-up

**Fig. 2:**
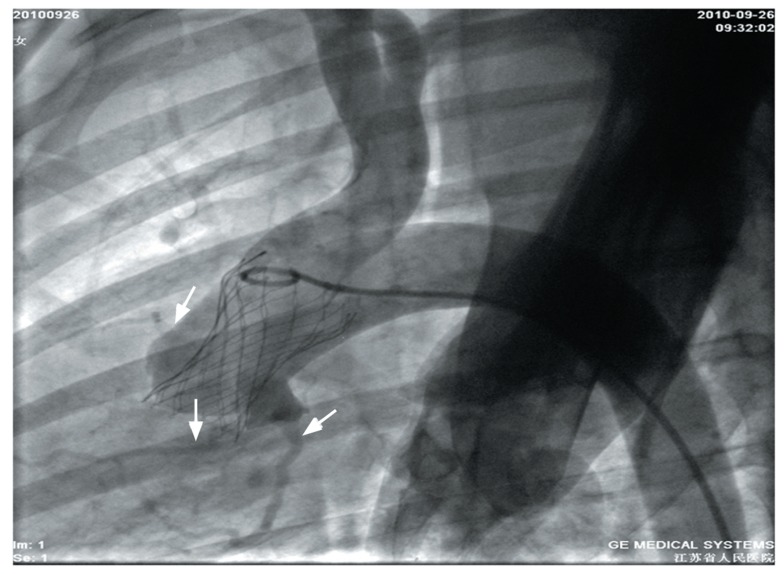
The supra-aortic angiogram after implantation of a valved stent. Angiography confirmed the well-anchored position of the device without evidence of aortic regurgitation or obstruction of coronary blood flow (arrows). In this case, we detected mild paravalvular aortic regurgitation after device deployment, but we selected this figure with no aortic regurgitation for clear visualization of coronary blood flow.

**Fig. 3:**
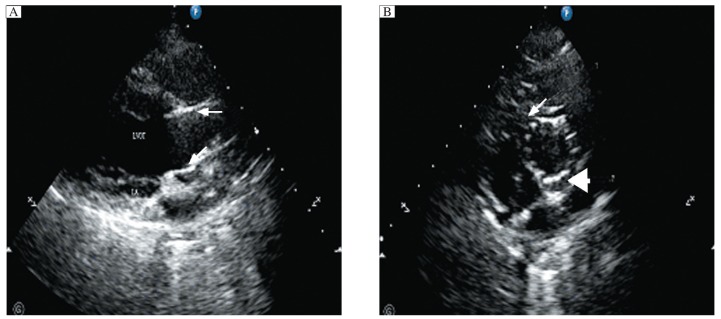
Echocardiographic image of a 23-mm device implanted into an aortic root with a 19.1-mm aortic annulus (2 h post procedure). Echocardiography showed that the device was in a good position and functioned normally. A: Long-axis, the stent (arrows) showed strong echo signals (LA=left atrium, LVOT=left ventricular outflow tract). B: Short-axis, the right coronary sinus (arrow) and left coronary sinus (arrowhead) were unaffected.

After device deployment, supra-aortic angiography was done immediately and 1 h later to determine the exact position and function of the implanted valve, and coronary blood flow. Trivial to mild aortic regurgitation appeared to be common in nearly all sheep. Blood flow in the coronary artery was confirmed to be normal by aortic angiography (***Fig. 2***). A TTE postprocedural test showed that the valve was in a good position and that the coronary sinus was unaffected (***Fig. 3A*** and ***3B***). No migration of the device was detected during follow-up. Hemodynamic parameters measured by echocardiography are shown in ***Table 2***. The repeated measures ANOVA confirmed that there was no statistical difference among time points in the LA diameter, LVDd, LVDs and LVEF (*P* > 0.05). Statistical differences were found in the mean transaortic gradient and the EOA. The ANOVA postestimation method (Wald test) was used for multiple comparisons which confirmed that, compared with baseline, the mean transaortic gradient increased and the effective orifice area decreased significantly immediately after procedure (*P* < 0.05), but no statistical difference was detected among three postprocedure timepoints (*P* > 0.05).

**Table 2:** Anatomic parameters and aortic regurgitation detected by echocardiography before, immediately, 1 and 2 h after the procedure

	Before (*n* = 10)	Immediately (*n* = 8)	1-h (*n* = 8)	2-h (*n* = 8)
Anatomic parameters				
LA diameter (mm)	21.20 ± 1.80	21.40 ± 1.50	21.20 ± 1.90	22.10 ± 2.10
LVDd (mm)	36.00 ± 2.20	36.30 ± 0.80	36.30 ± 1.20	37.80 ± 1.00
LVDs (mm)	20.80 ± 2.10	21.40 ± 1.60	20.60 ± 1.10	21.20 ± 2.30
LVEF	0.64 ± 0.04	0.60 ± 0.05	0.62 ± 0.07	0.66 ± 0.05
Mean transaortic gradient (mmHg)	2.18 ± 0.18	10.60 ± 2.30*	10.50 ± 1.70*	10.70 ± 1.50*
EOA (cm^2^)	2.60 ± 0.40	2.10 ± 0.30^#^	2.10 ± 0.20^#^	2.00 ± 0.20^#^
Aortic regurgitation^a^				
None	8(80.0%)	1(12.5%)	1(12.5%)	2(25.0%)
Trivial	1(10.0%)	4(50.0%)	4(50.0%)	3(37.5%)
Mild	1(10.0%)	2(25.0%)	2(25.0%)	2(25.0%)
Moderate	0	1(12.5%)	1(12.5%)	1(12.5%)
Severe	0	0	0	0

*Compared with before the procedure, the mean transaortic gradient increased significantly immediately, 1 and 2 h after the procedure, *P* < 0.05;

^#^Compared with before the procedure, the effective orifice area decreased significantly immediately, 1 and 2 h after the procedure, *P* < 0.05. ^a^Compared with baseline (before procedure), the AR level increased significantly immediately after procedure (*P* < 0.05), while there was no statistical difference among three post-procedure time points. EOA: effective orifice area; LA: left atrium; LVDd, diameter of the left ventricle end-diastolic period; LVDs: diameter of the left ventricle i end-systolic period; LVEF: left ventricular ejection fraction.

Immediately after stent deployment, 7 sheep (87.5%) had aortic regurgitation of different degrees whereas only 2 sheep (20%) had aortic regurgitation before the procedure (***Table 2***). In all 8 sheep with successful valve implantation, 5 sheep (62.5%) did not have or had trivial aortic regurgitation, 2 cases (25%) had mild aortic regurgitation, and only 1 sheep (12.5%) had moderate aortic regurgitation. Severe aortic regurgitation was not observed. The repeated measure ANOVA confirmed that the AR levels were found to be statistically different among four time-points, and subsequent multiple comparisons showed that, compared with baseline (before procedure), AR increased significantly immediately after procedure (*P* < 0.05), but there was no statistical difference concerning the three time points post procedure (*P* > 0.05). There was no association between the degree of valve oversize and the occurrence or degree of aortic regurgitation after TAVI (***Table 3***). The ratio of valve size/diameter of the aortic annulus in cases without or with trivial aortic regurgitation was similar to that with mild or moderate aortic regurgitation (1.19±0.06/1.17±0.07, *P* = 0.629). When considering the regurgitation type, the ratio of valve size/diameter of the aortic annulus in the paravalvular leak group was significantly higher than that in the valvular leak group (1.24±0.03 *vs*. 1.13±0.01, *P* = 0.003).

**Table 3:** Prosthesis sizing and aortic regurgitation immediately after the procedure

Subgroup	Diameter of the aortic annulus (mm)	Valve size/aortic annulus diameter ratio
Degree of aortic regurgitation (*n* = 8)		
None or trivial [5(62.5%)]	19.82 ± 1.76	1.19 ± 0.06
Mild or moderate [3(37.5%)]	19.80 ± 1.22	1.17 ± 0.07
Type of regurgitation (*n* = 7)		
Paravalvular leak [4(57.1%)]	20.95 ± 1.11*	1.13 ± 0.01^#^
Transvalvular leak [3(42.9%)]	18.47 ± 0.60	1.24 ± 0.04

*Compared with the transvalvular leak group, *P* = 0.018; ^#^Compared with the transvalvular leak group, *P* = 0.003.

**Fig. 4:**
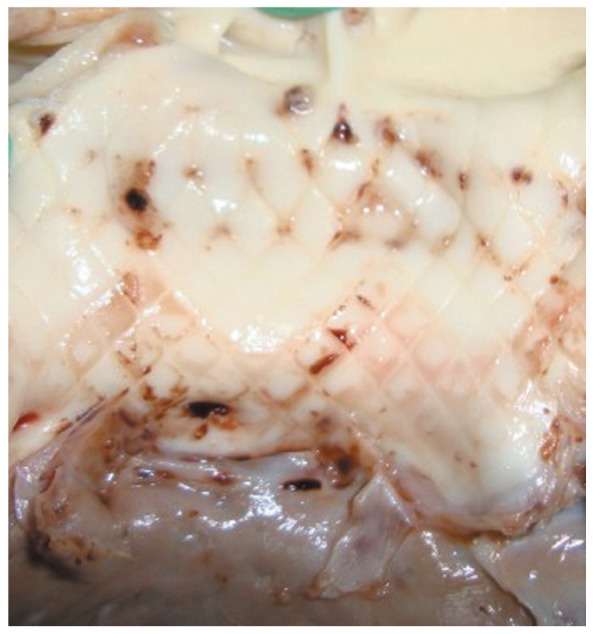
Gross anatomy after successful implantation of the device. The stent “fingerprint” on the aortic wall indicated high radial expansion of the stent.

Additionally, 2 h after implantation, no aortic regurgitation was observed in experimental animals. Only 1 sheep was detected with occasional ventricular premature. AV block was not detected. Autopsy findings 2 h after successful intervention showed that the device was well “anchored” against the aortic wall with its lower edge near the mitral valve annulus and yet far from the papillary muscles. The native aortic valves were placed aside, between the stent and the annulus. When the stent was removed, the fingerprint on the aortic intima was shown, indicating high radial expansion forces of the stent (***Fig. 4***). The orifice of the coronary artery and ascending aorta was not affected by the stent.

**Fig. 5:**
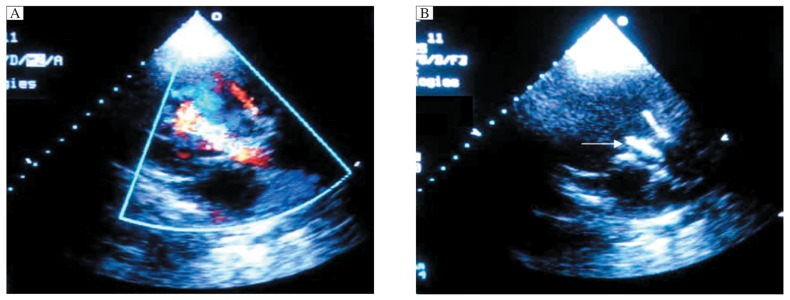
Echocardiographic image of moderate paravalvular aortic regurgitation after a 20-mm bioprothesis into an aortic root with a 19.6-mm native aortic annulus (long-axis, 2 h post procedure). A: An increased trans-aortic gradient (15.1 mmHg *vs* 3.6 mmHg pre-procedure) and moderate paravalvular aortic regurgitation were observed. B: Echocardiography showed that there was a gap between the device and the left ventricular outflow wall (arrow).

A deep fingerprint (indicating high radial forces) was detected on the left ventricular outflow wall. We therefore implanted a 20-mm bioprosthesis into a sheep with a 19.6-mm aortic annulus to ascertain if a smaller-size device with relatively lower radial forces could satisfy hemodynamic function. After successful implantation of the device, an increased trans-aortic gradient (15.1 mmHg versus 3.6 mmHg pre-procedure) and moderate paravalvular aortic regurgitation were observed (***Fig. 5A***). Echocardiography also confirmed that there was a gap between the lower portion of the device and the left ventricular outflow wall (***Fig. 5B***). The position of the device and hemodynamic parameters remained unchanged at 1 and 2 h follow-up.

## DISCUSSION

In recent years, TAVI has become an alternative to treat subjects with severe AS denied or not offered surgery due to high surgical risk or inoperability. Its feasibility and safety have been evaluated for nearly two decades[8]-[12]. However, some problems associated with the procedure (e.g., concomitant arterial conditions of elderly patients which make the intervention troublesome), some complications after the procedure, and the steep learning curve[13],[14] need addressing. To improve the procedure, we developed a new self-expanding revalving system. Compared with the traditional self-expanding aortic valve of identical size, the new revalving system had three characteristics. Firstly, the end part of the lower portion of our device had a smaller diameter, which may have alleviated radial pressures on sub-aortic tissue while avoiding migration after deployment. With a relatively smaller diameter of the lower portion, the valve could be delivered with a thinner and more flexible catheter (16 F). Secondly, the upper one-third of our device is 2 mm larger than Medtronic, which may provide bigger radial force for the stability of the device *in vivo*. Thirdly, the three T-shape claws on the upper end of the device can facilitate the loading of the device with corresponding T-shape sockets on the delivery cable.

In the present study, we validated the feasibility of the newly developed device by successfully implanting a 23-mm bioprosthesis in 8 sheep with a 16-F catheter. A thinner catheter may promise less injury to arteries and better manipulability. Hence, we hope that the newly developed device can make the procedure much safer and available to more elderly patients while smoothing the steep learning curve of this procedure. In most animal tests, the antegrade route is employed due to a larger catheter size or acute angle of the aortic arch of the animal[8]-[12]. In the present study, we successfully employed a retrograde route to implant the device. At the beginning of this study, we carried out pilot studies with pigs but found it very difficult to deliver the device to the aorta. The short carotid artery of the pig made delivery from the right common carotid artery difficult, whereas the slim arteries and acute angle of the aortic arch made delivery *via* the femoral artery even more difficult. Hence, we chose sheep as experimental animals to test our device system. Since the femoral arteries and iliac arteries of the sheep are too thin for the delivery system to pass through, we choose the right common carotid artery as the access of the retrograde route. Yet we should pay attention to the fact that the carotid artery is not the commonly employed access in clinical practice, so this pilot animal test could not mimic clinical scenario completely. Besides, due to the findings of significant variance in aortic anatomic data among sheep of identical weight, echocardiography or angiography should be employed to choose device size more precisely.

With respect to device positioning, it has been proposed that positioning of a valve in sheep is much more difficult than in pigs or humans because the distance between the coronary ostium and native aortic annulus is too short[15]. In our animal study, failure of the procedure has been caused by inappropriate positioning of the device, which relates to the techniques of the operators rather than the design of the device. Two h after the procedure, device migration was not observed. It has been reported that, as a key feature of procedural success, device positioning is primarily dependant upon the radial forces of the lower portion of the device against the native aortic annulus[13],[14],[16]. Interestingly, with the 20-mm bioprothesis, the device anchored against the aortic complex with only its upper portion pressing against the ascending aorta wall, whereas its lower portion was away from the left ventricular outflow wall. 1 and 2 h after the procedure, the position of the device was confirmed to be stable by angiography and echocardiography. This suggests that the radial forces of the lower portion are not vital in positioning of the device, as postulated previously.

To evaluate the safety of the implanted device, we detected the hemodynamic parameters of cases with successful implantation immediately, 1 and 2 h after the procedure. There was no significant decline in LVEF, LA diameter, and LVDd after the procedure. Compared with pre-procedure values, there was a significant increase in the mean transaortic gradient and decrease in the effective orifice area post the procedure, but it was within normal limits[7]. This may be from the acceleration in blood flow during systole after device implantation.

Though the association between aortic valvular leaks and device size was not confirmed in the present study, the type of aortic valvular leak was related to the ratio of valve size/diameter of the aortic annulus. The ratio was statistically larger in the transvalvular leak group compared with that in the paravalvular leak group. In the transvalvular leak group, inappropriate folding of the oversized valves confined to the relatively small aortic annulus led to intravalvular leaks. In the paravalvular leak group, although the valve unfolded sufficiently, the relatively smaller valve could not adhere completely to the wall of the aortic annulus, and leaks were found between the wall and the device. In subsequent study of implantation of the 20-mm device, moderate paravalvular aortic leaks were apparent between the device and the aortic wall, with a ratio of the valve size/aortic-annulus diameter of 1.02.

In the present study, an AV block was not detected immediately after device release and monitoring at 2 h. As a major and severe complication of TAVI, studies have reported that pacemaker implantation after complete AV block post procedure is primarily due to mechanical pressure and tissue injury derived from the implanted valve on the conductive bundle[6],[17]-[20]. Two risk factors deserve attention: implantation of a self-expanding bioprosthesis[6],[17]-[19] (compared with implantation of a balloon-expandable bioprosthesis, OR=3.781) and the occurrence of complete AV block immediately after device deployment[6]. The AV node is located in close proximity to the sub-aortic region and membranous septum of the ventricular outflow tract, with its most radial force located in the lower one-third portion. Hence, a relatively deeper insertion into the left ventricular outflow tract of an implanted self-expanding valve may cause more bundle injury than a balloon-expandable valve. Complete AV block immediately after device deployment may be a marker of too high a pressure or severe tissue injury on the conductive bundle caused by the implanted valve. In the present study, we decreased the diameter of the lower one-third portion of the device to alleviate its pressure on subaortic tissue. No AV block immediately after device release and at 2 h monitoring post procedure indicated that our device, with its relatively smaller diameter of the lower one-third portion, may be a promising approach to reduce the occurrence of this complication without migration of the device after successful implantation.

Several important limitations of this pilot study should be acknowledged. Firstly, the small number of test animals was an obvious limitation. Secondly, the follow-up may have been too short to provide valid data on the durability of biological valves. Thirdly, although the sheep model used is similar to human anatomy, it differed in several critical features, such as acute angulation of the aortic arch, the shorter ascending aorta, as well as the shorter distance between the coronary ostia and native aortic annulus. Another limitation was that we employed the carotid artery as the access of the retrograde route to implant the valve, which is not common in clinical practice.

In summary, it is feasible to implant the new self-expanding aortic valve with a 16-F delivery system into sheep hearts via the retrograde route. Hemodynamic performance and positioning of the implanted device was stable 2 h after successful implantation.
